# Using dynamic pupillometry as a simple screening tool to detect autonomic neuropathy in patients with diabetes: a pilot study

**DOI:** 10.1186/1475-925X-9-26

**Published:** 2010-06-17

**Authors:** Giselle L Ferrari, Jefferson LB Marques, Rajiv A Gandhi, Simon R Heller, Fábio K Schneider, Solomon Tesfaye, Humberto R Gamba

**Affiliations:** 1 Electrical Engineering Department, Federal University of Paraná, Curitiba, PR - Brazil; 2 School of Electrical Engineering and Applied Computer Sciences-CPGEI, Federal University of Technology-Paraná, Curitiba, PR - Brazil; 3 Institute Biomedical Engineering, Electrical Engineering Department, Federal University Santa Catarina, Florianópolis, SC - Brazil; 4 Royal Hallamshire Hospital, University of Sheffield, Sheffield, UK

## Abstract

**Background:**

Autonomic neuropathy is a common and serious complication of diabetes. Early detection is essential to enable appropriate interventional therapy and management. Dynamic pupillometry has been proposed as a simpler and more sensitive tool to detect subclinical autonomic dysfunction. The aim of this study was to investigate pupil responsiveness in diabetic subjects with and without cardiovascular autonomic neuropathy (CAN) using dynamic pupillometry in two sets of experiments.

**Methods:**

During the first experiment, one flash was administered and the pupil response was recorded for 3 s. In the second experiment, 25 flashes at 1-s interval were administered and the pupil response was recorded for 30 s. Several time and pupil-iris radius-related parameters were computed from the acquired data. A total of 24 diabetic subjects (16 without and 8 with CAN) and 16 healthy volunteers took part in the study.

**Results:**

Our results show that diabetic subjects with and without CAN have sympathetic and parasympathetic dysfunction, evidenced by diminished amplitude reflexes and significant smaller pupil radius. It suggests that pupillary autonomic dysfunction occurs before a more generalized involvement of the autonomic nervous system, and this could be used to detect early autonomic dysfunction.

**Conclusions:**

Dynamic pupillometry provides a simple, inexpensive, and noninvasive tool to screen high-risk diabetic patients for diabetic autonomic neuropathy.

## Background

Diabetic autonomic neuropathy (DAN) is the least recognized and understood complications of diabetes, despite its significant negative impact on survival and quality of life in people with diabetes [1]. It can involve the entire autonomic nervous system. Among the most serious DAN is cardiovascular autonomic neuropathy (CAN), which encompasses damage to the autonomic nerve fibers that innervate the heart, affecting heart rate and blood pressure control and vascular dynamics. Diabetes-related CAN occur in one-quarter of the subjects with Type 1 diabetes and in one-third of those with Type 2 diabetes. CAN is associated with increased mortality, silent myocardial ischemia, and may even predict the development of stroke [2]. While DAN can be clinically evident, manifested by dysfunction of one or more organ systems (*e.g. *cardiovascular, gastrointestinal, genitourinary, sudomotor, or ocular) [3], it is frequently subclinical and asymptomatic.

A recent statement of the ADA [4] concerning diabetic neuropathies recommends that screening should be instituted at diagnosis of Type 2 diabetes and 5 years after the diagnosis of Type 1 diabetes. Screening might comprise a history and an examination for the signs of autonomic dysfunction. Earlier identification of these high-risk individuals is clearly important, as it would allow for the deployment of management strategies to improve long-term outcomes. If diagnosis is delayed until the development of symptoms, the outcome is extremely poor, with a 5-year survival rate of only 50% [5].

Although several conventional autonomic function tests exist to aid clinicians in the evaluation of DAN, they require specific equipments and well-trained personnel, and are time-consuming to perform [6]. Additionally, these tests typically require active patient participation and compliance, leading to their use in a minority of subjects, mainly those with advanced autonomic neuropathy who have already developed symptoms. Therefore, the development of new approaches for the early identification of DAN, i.e., before the onset of symptoms, is desired. This would allow for interventions and management that may result in reduced long-term complications, as previously reported [7]. In this context, simple and inexpensive approach to screen large number of subjects with diabetes for autonomic neuropathy would be valuable to minimize the enormous social and economic burden caused by DAN, especially for subjects at a much higher risk, such as those with undiagnosed CAN. The pupil dynamics analysis, i.e., dynamic pupillometry, has the potential for supporting such a screening approach.

Pupil size, shape, and reactivity to light have been used as indicators of neurological function in brain-injured patients, particularly in comatose patients. There are circular and radial muscles that control the size of the pupil. The former is innervated by parasympathetic fibers, and the latter, by sympathetic fibers. Thus, the pupil radius is controlled by both the sympathetic and parasympathetic autonomic nervous system in response to environmental light, a mechanism called the pupil light reflex. Therefore, the pupillary radius response to an external light stimulus might provide an indirect means to assess the integrity of neuronal pathways controlling pupil size [8] and an early indication of DAN [9].

Sympathetic stimulation of α-1 adrenergic receptors causes the contraction of the radial muscle, and subsequent dilatation of the pupil (mydriasis). Conversely, parasympathetic stimulation causes contraction of the circular muscle and constriction of the pupil (miosis). The pupillary reflex is mediated by acetylcholine and noradrenaline, causing miosis and mydriasis, respectively. Thus, change in pupil size in response to a light stimulus is based on a functional equilibrium between sympathetic and parasympathetic activity [10].

It is therefore reasonable to expect that generalized conditions of the autonomic system will affect the pupil response to the light stimulus [11]. Parasympathetic dysfunction might cause relative mydriasis of the pupil in light conditions and diminished constrictor reflexes with or without pupillotonia, which is thought to result from aberrant re-innervations. Sympathetic dysfunction might cause relative miosis of the pupil in the dark, increased re-dilatation lag, and attenuation of the startle reflex, as observed in Horner's syndrome [12]. Investigations in subjects with diabetes have revealed abnormal responses of the pupil-radius modulation in some subjects, especially in those with diabetic neuropathy [9,13-18], supporting the potential of pupillometry to be used as a screening tool for autonomic neuropathy in the diabetic population [15-17].

Previous studies have described the resting pupil radius as a sympathetic parameter, and the reflex amplitude as a parasympathetic parameter in healthy and diabetic subjects [9,13-18]. However, there is very little evidence with regard to the relationship between the pupil-iris ratio and other time-related parameters with autonomic neuropathy in diabetes subjects with and without CAN. In this study, the results of an investigative study of the pupil responsiveness to light flashes in healthy and diabetic subjects with and without clinical evidence of CAN has been presented. The study was performed with a custom-built dynamic pupillometer [19] that allows the computation of a larger number of parameters when compared with commercially available pupillometers.

## Methods

### Subjects

This study comprised three groups: Group 1 - 16 healthy volunteers (9 males and 7 females); Group 2 - 16 diabetic subjects without CAN (15 males and 1 females); and Group 3 - 8 diabetic subjects with CAN (7 males and 1 females). The study was conducted in the Diabetes Centre, Royal Hallamshire Hospital, University of Sheffield, UK.

Informed consent was obtained from all participants. Subjects were not receiving any drugs affecting sympathetic or parasympathetic pupillary function, and those with past history of ocular operations, nonsymmetrical pupil, misshapen pupil, or conditions affecting pupillary reflexes were excluded [20]. All diabetic subjects underwent standard blood and biochemical laboratory tests.

Owing to the experiment setup, only the right side of each participant was accessible for pupillometry. The pupil reaction was assessed twice: first with one flash, and 5 min later with 25 flashes. Both tests were performed in the right eye, and all the subjects were tested between 9 and 12 am. For all patients, at least 8 h of sleep was required in the preceding night without any hypoglycemic episode.

### The pupil stimulator and response recorder

**Figure 1:**
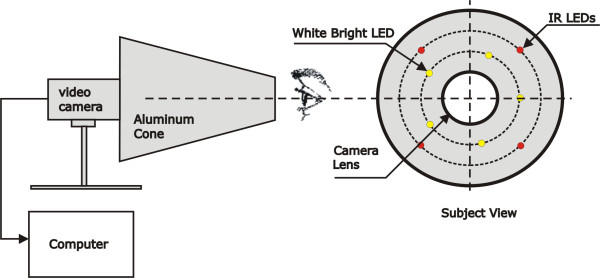
**Setup of the Pupil Stimulator and Response Recorder (PSRR) instrument developed to perform *in vivo *experiments**.

Figure 1 illustrates the setup of the instrument, named Pupil Stimulator and Response Recorder (PSRR), developed to stimulate and record the pupil response to flashes of white light [19]. It consisted of a commercial monochromatic CCD analog video camera (EIA-RS-170 standard) that captures an image frame every 1/30 seconds; four infrared (IR) light emitter diodes (LEDs) with a diameter of 5 mm; five high-intensity bright white LEDs with a diameter of 8 mm; a 17-cm height aluminum cone section with extremity diameters of 14.5 and 5.6 cm; an electronic current supply to the LEDs; a built unit for timing control based on the microcontroller PIC16F873 (Microchip Technology Inc., Arizona, USA); and a Pentium IV 3 GHz Notebook with a PCMCIA frame grabber to record and process the pupil images. The aluminum cone was fixed in a printed circuit board (that hold the IR and white LEDs), which was then fixed in the CCD camera. The IR and the high-intensity bright white LEDs were symmetrically placed around the CCD lens, as illustrated in Figure 1. The IR LEDs promoted the illumination with a high pupil to iris contrast, but without producing any pupil response. The lens focus was manually adjusted by rotating the aluminum cone. The pupil and the aluminum cone wall acted as a mirror, reflecting the IR LEDs light. The light reflection in the cone wall caused too many artifacts in the pupil image, making the pupil-iris edge detection difficult during the image processing phase. To avoid these artifacts, the aluminum cone interior was blackened.

The PSRR can stimulate the pupil with one flash or a series of 25 flashes (1-Hz frequency), with fixed intensity (250 cd) and duration (10 ms). The changes in the radius of the pupil were evaluated as a function of several time-dependent and pupil-iris radius ratio parameters.

The pupil radius was automatically determined for each frame in the recorded video stream. The offline image processing algorithm was implemented using MATLAB (The MathWorks Inc., Massachusetts, USA) environment and was divided into three major steps: elimination of the IR LEDs light reflex within the pupil; enhancement of the image borders; and pupil and iris edge detection.

**Figure 2:**
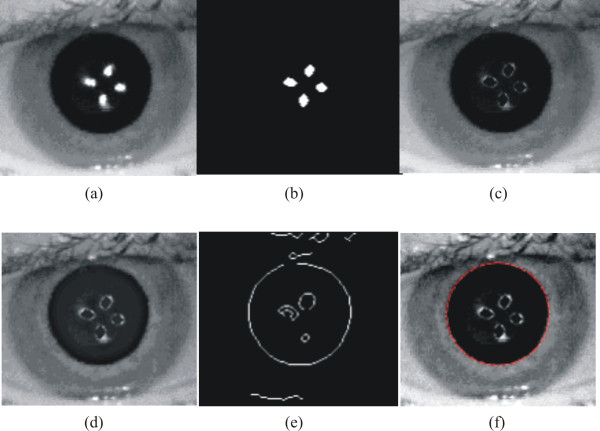
**Pupil radius measurement sequence**. (a) ROI selected manually by the user; the four brighter regions in the image correspond to the IR LEDs light reflected by the retina; (b) Image mask with pixels values set to white in the position of the IR LEDs light reflected; (c) the ROI in (a) with the corresponding white pixels in (b) set to a gray level of 35; (d) result of edge enhancement and scale transformed of (c); (e) pupil edge; and (f) the circle that gives the maximum correlation with the pupil border in (d) over the ROI processed image in (d).

Figure 2 shows the images obtained in some of the major algorithms steps until the best circle that fits the pupil is found. Figure 2(a) shows the region of interest (ROI) that is cropped from the original image. First, the IR LED light reflection was detected using the Canny edge detector with a threshold set to 0.8. The detector returned one pixel line that makes a frontier between the white reflected and the dark pupil regions. These contours were then filled, forming a reference mask to change the white pixel in the pupil by a gray value of 35. It is important to note that owing to our image acquisition system capturing the eye images under the same illumination condition, the pupil darkness of every subject has a gray value around 35. Figure 2(b) and 2(c) shows the mask and the eye image with the IR LED light reflection removed. This image was then edge enhanced using a high-boost filtering [21], followed by an intensity transformation through a 0.6-gamma function. The result is shown in Figure 2(d). A Canny edge detector with a 0.5-threshold was then applied to find the frontier pixels between the pupil and iris, as shown in Figure 2(e). To determine the pupil radius, it was found that the best solution was by convolving the image shown in Figure 2(e) with contour circle masks with different radius (20-90 pixels, with 0.2 pixels of resolution), and determining the circle radius that gives the maximum correlation.

**Figure 3:**
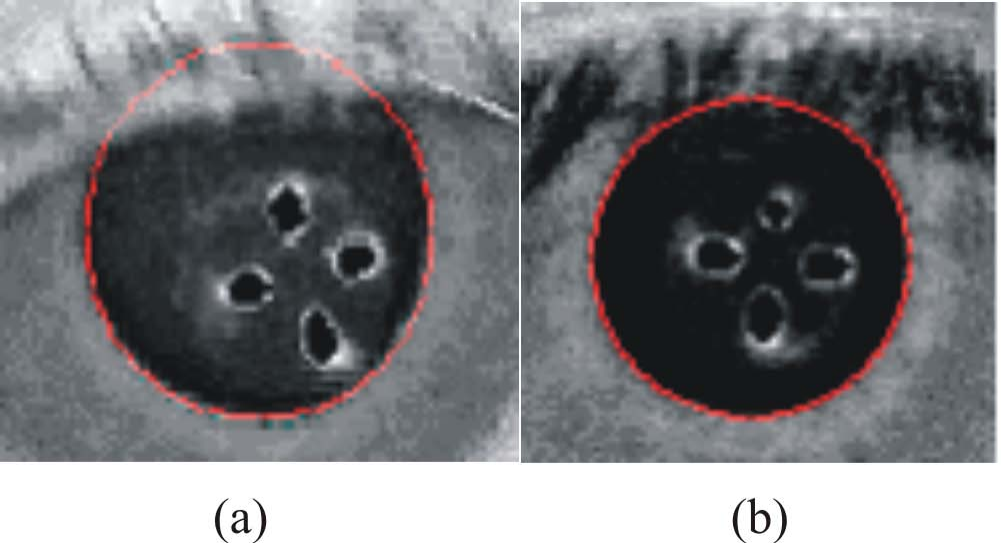
**Pupil-radius determination with the eyelid (a) and eyelashes (b) over the pupil**.

This algorithm was even able to determine the pupil radius in those cases where the pupil was under the eyelid or eyelashes, as shown in Figure 3(a) and 3(b).

The iris radius was automatically determined for each frame in the recorded video stream using the Hough Transform, followed by a canny edge detector algorithm [21].

### Experiments

All the subjects underwent two experiments. In the first experiment, the subject dark-adapted for 2 min leaning on the cone before a single light flash was administered. The pupil response was recorded for 3 s. During this period, the subject was instructed to avoid or minimize blinking.

In the second experiment, the subject dark-adapted for 2 min before 25 flashes were administered at 1-s interval. The pupil response was recorded for 30 s. In both the experiments, the other eye was covered with a black fabric to avoid any external light interference.

#### Single light flash parameters

**Figure 4:**
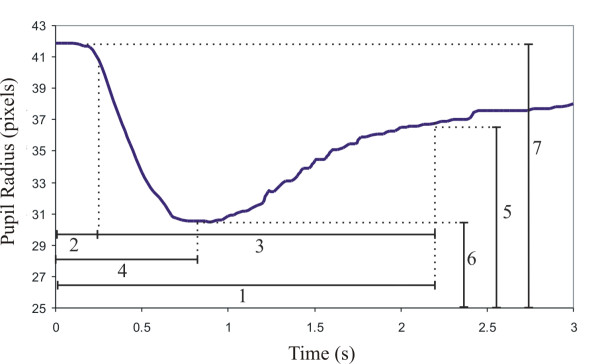
**Typical pupil reflex of a healthy volunteer after a 10-ms light flash intensity of 250 cd was triggered at zero second**. The indicated parameters are defined as follows: 1-Latency time to reach the plateau at 75% of pre-flash pupil radius; 2-Latency time to the beginning of constriction; 3-Duration of constriction; 4-Latency to the largest constriction; 5- Radius of pupil on reaching the plateau; 6-Radius of pupil at largest constriction; 7- Radius of pupil before flash.

Figure 4 shows a typical pupil response obtained from a healthy volunteer. It shows the radius of the pupil in each captured frame in pixels as a function of time.

The pupil reflex evaluation parameters can be defined as follows [10]:

• Pupil-iris radius ratio in the frame preceding the flash, #7 in Figure 4, divided by iris radius;

• Latency from flash exposure to the start of constriction (when pupil radius decreases to 90% of pre-flash value), #2 in Figure 4;

• Pupil-iris radius ratio (#6 in Figure 4 divided by iris radius) and latency (#4 in Figure 4) to the smallest size of the pupil;

• During the recovery phase: pupil-iris radius ratio (#5 in Figure 4 divided by iris radius) and latency to plateau at 75% of pre-flash pupil radius (#1 in Figure 4);

• Duration of constriction (#3 in Figure 4);

• Reflex amplitude - resting pupil radius (#7 in Figure 4) minus minimum pupil radius after the light stimulus (#6 in Figure 4);

• Velocity of constriction - changing rate in the pupil radius over the reflex amplitude time interval: reflex amplitude divided by #3 in Figure 4.

#### Twenty-five light flashes parameters

**Figure 5:**
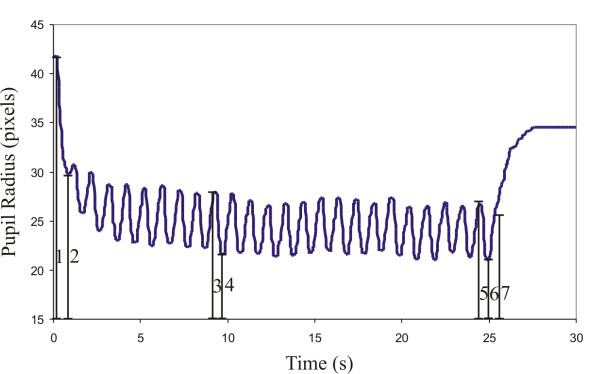
**Typical pupil reflex of a healthy volunteer in the experiment with a series of 25 flashes at 1-s intervals**. The indicated parameters are defined as follows: 1- Radius of pupil before the first flash; 2- Radius of pupil at largest constriction after the first flash; 3- Radius of pupil before the 10^th ^flash; 4- Radius of pupil at largest constriction after the 10^th ^flash; 5- Radius of pupil before the 25^th ^flash; 6- Radius of pupil at largest constriction after the 25^th ^flash; 7-Radius of pupil on reaching the plateau at 75% of pupil radius preceding the 25^th ^flash.

Figure 5 shows a typical pupil response obtained from a healthy volunteer. It shows the radius of the pupil in pixels for each captured frame as a function of time and the main parameters measured.

For the 25-flashes experiment, the pupil reflex evaluation parameters can be defined as follows [10]:

• Pupil-iris ratio in the frame preceding the 10^th ^(#3 in Figure 5 divided by iris radius) and 25^th ^flash (#5 in Figure 5 divided by iris radius);

• Pupil-iris ratio of the smallest size of the pupil after the 10^th ^(#4 in Figure 5 divided by iris radius) and 25^th ^flash (#6 in Figure 5 divided by iris radius);

• Latency from flash exposure to the start of constriction after the 10^th ^and 25^th ^flash;

• Latency to the smallest size of the pupil after the 10^th ^and 25^th ^flash;

• During the recovery phase: pupil-iris ratio (#7 in Figure 5 divided by iris radius) and latency to plateau when the pupil radius reaches 75% of the pre-flash value;

• Duration of constriction.

### Assessment for CAN

A standardized set of cardiovascular tests was used to assess the prevalence of CAN. The tests were performed after a resting period of 10 min to ensure that the heart rate was at its baseline values. The tests included recording the electrocardiogram (ECG) to determine the heart rate variability (HRV), and measurements of systolic and diastolic blood pressure (BP). Four tests of HRV and one test of BP control were performed and evaluated according to standard criteria [22]. Recordings were carried out at rest (HRV and BP), deep breathing (HRV), Valsalva maneuver (HRV), and active standing up (HRV and BP). The total duration of these tests was approximately 30 min. Diabetic subjects were classified as having CAN if the results of two or more of these five autonomic function tests were below age-adjusted normative values [22].

### Statistical analysis

Statistical analysis for all the variables included the calculation of mean and standard deviation (SD). One-way ANOVA and post-hoc Bonferroni statistical tests were used to compare the pupillography results between the groups. Comparisons involving the diabetes group (without CAN vs. with CAN) were carried out using Kruskal-Wallis Test. SPSS version 15.0 (SPSS Inc., Chicago, USA) was used to analyze the data, and *p *values less than 0.05 were considered statistically significant.

## Results

### Single light flash experiment

The mean and SD values for each parameter used to evaluate the response of the pupil reaction to a single flash and the statistical significance tests are shown in Table 1.

**Table 1:** Subjects' baseline characteristics and pupillometry parameters

Parameters	Healthy Volunteers	Diabetes		*p *values		
		Without CAN	With CAN	***p***_**1**_	***p***_**2**_	***p***_**3**_
Number of subjects	16	16 (9 Type 1, 7 Type 2)	8 (3 Type 1, 5 Type 2)	-	-	-

Gender (M/F)	9/7	15/1	7/1	-	-	-

Age (years)	44 ± 13	50 ± 11	51 ± 5	ns	ns	ns

Duration of diabetes (years)	-	20 ± 10	22 ± 10	-	-	ns

HbA1c (%)	-	8.3 ± 1.1	9.4 ± 2.2	-	-	ns

Systolic blood pressure (mmHg)	120 ± 13	141 ± 20	147 ± 16	*	*	ns

Diastolic blood pressure (mmHg)	74 ± 6	77 ± 9	87 ± 10	ns	*	ns

Ratio P/I in darkness	0.55 ± 0.056	0.42 ± 0.045	0.35 ± 0.077	$	$	*
Latency to constriction (s)	0.20 ± 0.078	0.26 ± 0.070	0.29 ± 0.100	ns	*	ns
Ratio P/I at largest constriction	0.40 ± 0.050	0.30 ± 0.031	0.26 ± 0.065	$	$	ns
Latency to largest constriction (s)	0.90 ± 0.147	1.01 ± 0.192	0.91 ± 0.089	ns	ns	ns
Reflex amplitude (pixels)	9.62 ± 1.99	7.40 ± 1.90	5.35 ± 2.14	#	#	ns
Ratio P/I of plateau	0.50 ± 0.055	0.37 ± 0.046	0.32 ± 0.068	$	$	ns
Latency to plateau (s)	1.88 ± 0.539	1.95 ± 0.744	1.49 ± 0.331	ns	ns	ns
Duration of constriction (s)	1.63 ± 0.559	1.69 ± 0.75	1.19 ± 0.318	ns	ns	ns
Velocity of constriction (pixels/s)	14.57 ± 5.32	10.28 ± 3.49	7.87 ± 3.48	*	#	ns

[mean ± standard deviation] for the single flash experiment. *p*_1 _- Comparison between healthy volunteers and diabetic subjects without CAN. *p*_2 _- Comparison between healthy volunteers and diabetic subjects with CAN. *p*_3 _- Comparison between diabetic subjects without and with CAN. ns - not significant, * - *p *< 0.05, # - *p *< 0.01, and $ - *p *< 0.001.

There was no significant difference between the three groups with regard to age (*p *> 0.05). The duration of diabetes did not differ in the diabetic subjects with (22 ± 10 years [mean ± SD]) and without CAN (20 ± 10 years), and HbA1c (%) was not significantly different between these two groups (9.4 ± 2.2 and 8.3 ± 1.1, respectively).

Systolic blood pressure was significantly lower (*p *< 0.05) in healthy volunteers (120 ± 13 mmHg) when compared with diabetic subjects without CAN (141 ± 20 mmHg) and diabetic subjects with CAN (147 ± 16 mmHg). Moreover, diastolic blood pressure was significantly greater (*p *< 0.05) in diabetic subjects with CAN (87 ± 10 mmHg) than in diabetic subjects without CAN (77 ± 9 mmHg).

**Figure 6:**
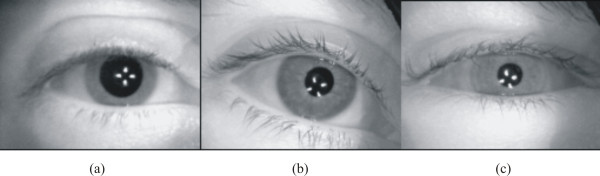
**Recorded IR-illuminated images of the eye of three subjects showing the pupil at its maximum mydriasis for (a) healthy volunteer, (b) diabetic subject without CAN, and (c) diabetic subject with CAN**.

Table 1 indicates that the pupil-iris ratio in the frame preceding the light flash was significantly greater (*p *< 0.001) in healthy volunteers (0.55 ± 0.056 [mean+SD]) than in diabetic subjects without CAN (0.42 ± 0.045) and diabetic subjects with CAN (0.35 ± 0.077). The remarkable difference in the pupil size in darkness can be seen in Figure 6 which shows the images of a healthy volunteer (a), diabetic subject without CAN (b), and diabetic subject with CAN (c). In Figure 6, the four white spots within the pupil are the reflecting IR LEDs used to illuminate the eye during image recording.

Latency from flash exposure to the start of constriction was significantly longer (*p *< 0.01) in the diabetic subjects with CAN (0.29 ± 0.100 s) than in healthy volunteers (0.20 ± 0.078 s).

Similarly, the pupil-iris ratio in the frame of largest constriction was significantly greater (*p *< 0.001) in healthy volunteers (0.40 ± 0.050) than in diabetic subjects without CAN (0.30 ± 0.031) and with CAN (0.26 ± 0.065).

However, the latency to the largest constriction of the pupil was not significantly different between the groups.

**Figure 7:**
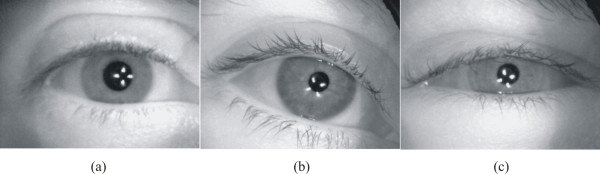
**Recorded IR-illuminated images of the eye of three subjects showing the pupil at its maximum miosis for (a) healthy volunteer, (b) diabetic subject without CAN, and (c) diabetic subject with CAN**.

The difference in the pupil size in the frame of largest constriction is illustrated in Figure 7 which shows the images of healthy volunteer (a), diabetic subject without CAN (b), and diabetic subject with CAN (c).

During the recovery phase, pupil-iris ratio in the plateau was significantly greater (*p *< 0.001) in healthy volunteers (0.50 ± 0.055) than in diabetic subjects with CAN (0.32 ± 0.068) and diabetic subjects without CAN (0.37 ± 0.046).

No significant differences were found between the groups in the latency to reach the plateau and duration of constriction.

Reflex amplitude measured in pixels was significantly higher (*p *< 0.01) in healthy volunteers (9.62 ± 1.99) when compared with diabetic subjects without CAN (7.40 ± 1.90) and diabetic subjects with CAN (5.35 ± 2.14). Moreover, the velocity of constriction (pixels/s) was significantly higher (*p *< 0.05) in healthy volunteers (14.57 ± 5.32) when compared with diabetic subjects without CAN (10.28 ± 3.49) and diabetic subjects with CAN (7.87 ± 3.48).

No significant difference was found between the diabetic groups, with exception for the P/I ratio in darkness.

**Figure 8:**
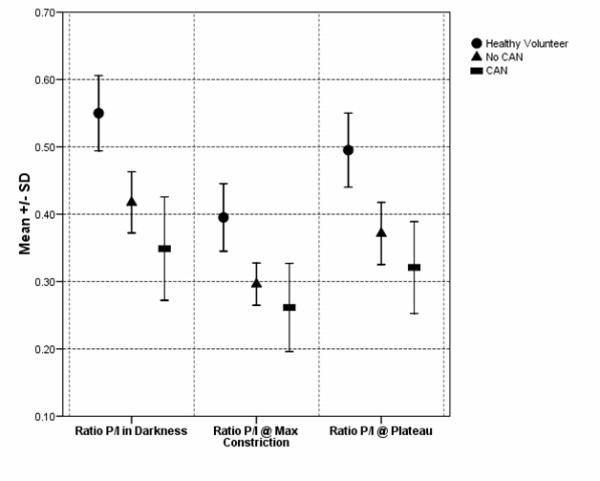
**Comparison of mean and standard deviation values of the pupil-iris ratio (P/I) in the largest mydriasis before flash (#7 in Figure 4), at largest miosis (#6 in Figure 4), and during the plateau period (#5 in Figure 4)**. These values were obtained after one 10-ms light flash in healthy volunteers and diabetic subjects with and without CAN.

Figure 8 shows a comparison of the mean and SD of the pupil-iris ratios among the three groups.

### Twenty-five light flashes experiment

In the second experiment, one diabetic subject without CAN and three diabetic subjects with CAN blinked more than three times during the experiment and were excluded.

There was no significant difference between the three groups with regard to age (*p *> 0.05). The duration of diabetes did not differ in the diabetic subjects with (20 ± 15 years) and without CAN (20 ± 11 years), and HbA1c (%) was not significantly different between these two groups (10.2 ± 2.1 and 8.3 ± 1.1).

Systolic blood pressure was significantly lower (*p *< 0.05) in healthy volunteers (120 ± 13 mmHg) when compared with diabetic subjects without CAN (143 ± 20 mmHg) and diabetic subjects with CAN (145 ± 14 mmHg). Moreover, diastolic blood pressure was significantly greater (*p *< 0.05) in diabetic subjects with CAN (86 ± 8 mmHg) than in diabetic subjects without CAN (78 ± 9 mmHg).

Table 2 shows the mean and SD values of the calculated parameter used to evaluate the response of the pupil reaction to 25 flashes and the statistical significance tests.

**Table 2:** Subjects' baseline characteristics and pupillometry parameters

Flash	Parameters	Healthy Volunteers	Diabetic		*p *values		
			
			Without CAN	With CAN	***p***_**1**_	***p***_**2**_	***p***_**3**_
	Number of subjects	16	15 (9 Type 1, 7 Type 2)	5 (2 Type 1, 3 Type 2)	-	-	-

	Gender (M/F)	9/7	14/1	4/1	-	-	-

	Age (years)	44 ± 13	51 ± 11	49 ± 4	ns	ns	ns

	Duration of diabetes (years)	-	20 ± 11	20 ± 15	-	-	ns

	HbA1c (%)	-	8.3 ± 1.1	10.2 ± 2.1	-	-	ns

	Systolic blood pressure (mmHg)	120 ± 13	143 ± 20	145 ± 14	*	*	ns

	Diastolic blood pressure (mmHg)	74 ± 6	78 ± 9	86 ± 8	ns	*	ns

**10**^**th**^	Ratio P/I preceding the 10^th ^flash	0.33 ± 0.050	0.26 ± 0.024	0.28 ± 0.071	$	ns	ns
	Latency to constriction (s)	0.35 ± 0.139	0.42 ± 0.126	0.44 ± 0.192	ns	ns	ns
	Latency to largest constriction (s)	0.68 ± 0.165	0.75 ± 0.133	0.68 ± 0.293	ns	ns	ns
	Ratio P/I at largest constriction	0.28 ± 0.049	0.24 ± 0.021	0.27 ± 0.076	*	ns	ns

**25**^**th**^	Ratio P/I preceding the 25^th ^flash	0.32 ± 0.044	0.26 ± 0.022	0.30 ± 0.062	#	ns	ns
	Latency to constriction (s)	0.31 ± 0.125	0.36 ± 0.070	0.40 ± 0.155	ns	ns	ns
	Ratio P/I at largest constriction	0.27 ± 0.046	0.23 ± 0.020	0.25 ± 0.063	ns	ns	ns
	Latency to largest constriction (s)	0.69 ± 0.123	0.72 ± 0.054	0.79 ± 0.138	ns	ns	ns
	Ratio P/I of plateau	0.30 ± 0.041	0.25 ± 0.021	0.28 ± 0.060	$	ns	ns
	Latency to plateau (s)	0.95 ± 0.168	0.95 ± 0.082	1.35 ± 0.583	ns	#	#
	Duration of constriction (s)	0.64 ± 0.237	0.59 ± 0.145	0.95 ± 0.675	ns	ns	ns

[mean ± standard deviation] for the 25 flashes experiment. *p*_1 _- Comparison between healthy volunteers and diabetic subjects without CAN. *p*_2 _- Comparison between healthy volunteers and diabetic subjects with CAN. *p*_3 _- Comparison between diabetic subjects without and with CAN. ns - not significant, * - *p *< 0.05, # - *p *< 0.01, and $ - *p *< 0.001.

Pupil-iris ratio in the frame preceding the 10^th ^flash was significantly greater (*p *< 0.001) in healthy volunteers (0.33 ± 0.050) than in diabetic subjects without CAN (0.26 ± 0.024), but no significant difference was found between the healthy volunteers and diabetic subjects with CAN.

The latency from the 10^th ^flash exposure to the start of constriction was not significantly different between the groups.

There was no difference between the groups in latency to the largest constriction of the pupil after the 10^th ^flash.

The pupil-iris ratio to the largest constriction was significantly greater (*p *< 0.05) in healthy volunteers (0.28 ± 0.049) than in diabetic subjects without CAN (0.24 ± 0.021), but no significant difference was found between the healthy volunteers and diabetic subjects with CAN.

For the 10^th ^flash, no significant difference was found between diabetic groups.

**Figure 9:**
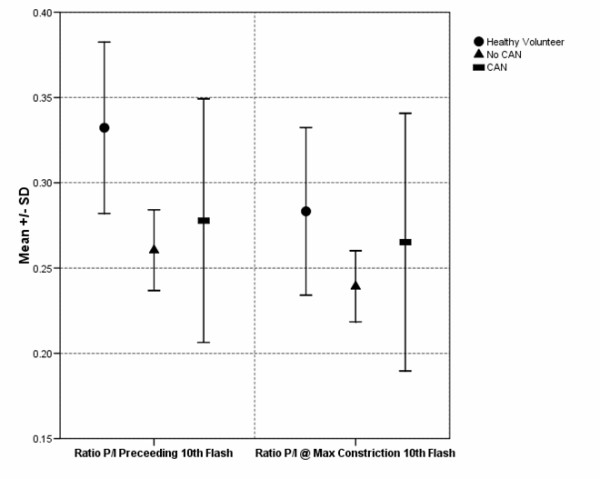
**Comparison of mean and standard deviation values of the pupil-iris ratios (P/I) before the 10^th ^flash (#3 in Figure 5) and at the largest constriction (#4 in Figure 5)**.

The significant differences between groups, in pupil-iris ratios at 10^th ^flash for the second experiment, are illustrated in Figure 9.

The pupil-iris ratio one frame before the 25^th ^flash was also significantly greater (*p *< 0.01) in healthy volunteers (0.32 ± 0.044) than in those diabetic subjects without CAN (0.26 ± 0.022), but no significant difference was found between the healthy volunteers and diabetic subjects with CAN.

There were no significant differences between the three groups in latency to constriction at the 25^th ^flash exposure, pupil-iris ratio, and latency to the largest constriction of the pupil after the 25^th ^flash.

During the recovery phase, the pupil-iris ratio at plateau was significantly greater (*p *< 0.001) in healthy volunteers (0.30 ± 0.041) than in diabetic subjects without CAN (0.28 ± 0.060), but no significant difference was found between the healthy volunteers and diabetic subjects with CAN.

Moreover, the latency to plateau was significantly shorter (*p *< 0.01) in healthy volunteers (0.95 ± 0.168 s) than in diabetic subjects with CAN (1.35 ± 0.583 s).

**Figure 10:**
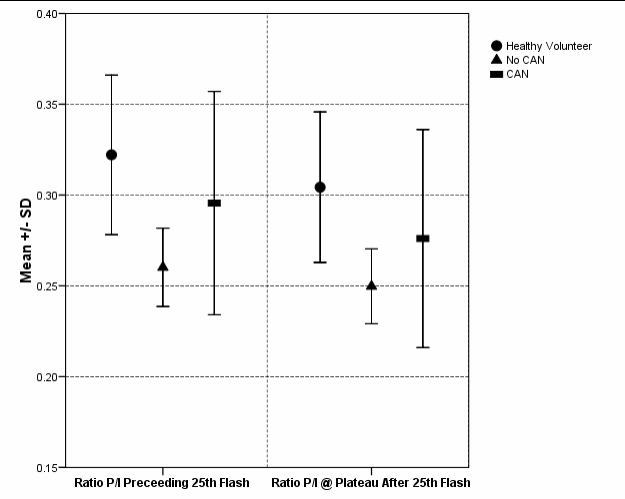
**Comparison of mean pupil-iris ratios (P/I) preceding 25^th ^flash (#5 in Figure 5) and during the plateau period (#7 in Figure 5) in healthy volunteers (HV) and diabetic subjects with and without CAN (Error bars represent SD)**.

Figure 10 shows a comparison of the mean and SD of the pupil-iris ratios at the 25^th ^flash among the three groups.

Duration of the constriction was not significantly different between the three groups.

For the 25^th ^flash, no significant difference was found between the diabetic groups, with an exception in the latency to plateau.

## Discussion

The results of the first experiment confirm the findings of previous studies [9,12-15] of a smaller resting pupil size and smaller reflex amplitude in subjects with diabetes.

According to Smith and Dewhirst [18], the resting pupil size is mainly under sympathetic control and radius reduction is a sign of diminished sympathetic outflow to the iris muscles. During the constriction phase, radius and time parameters mainly reflect parasympathetic function. However, both the systems are active during the recovery phase [15].

Smith and Smith [13] concluded that decreased reflex amplitudes in diabetic subjects cannot be ascribed to small pupil radius at rest, but are owing to a parasympathetic dysfunction. From our results in Table 1 comparing healthy volunteers and diabetic subjects (with and without CAN), a significant reduction in the pupil radius at baseline and amplitude of the pupil reflexes was found in both the diabetic groups. This might indicate that both sympathetic and parasympathetic dysfunction is affecting the pupillary reflex in these groups.

The significantly smaller initial pupil size seen in the diabetes group without CAN when compared with healthy subjects might be regarded as an early sign of involvement of the autonomic nerve system before cardiac manifestation of systemic autonomic neuropathy [9].

With regard to the second experiment, no comparison with previous findings was possible because no similar studies have been reported in the literature. The second experiment was found to lead to a gradual increase in the parasympathetic tone as a result of repeated light stimulation. This condition provides a different level of balance between parasympathetic and sympathetic nervous system, which is not linearly correlated to those of the first experiment. Although the two components of the autonomic nervous system may have abnormal tones, their combination may produce normal results during the first experiment. Moreover, the pupil muscles may perform well during a single flash administration, but this may not be true for repeated stimulation. The second experimental condition provided data on the flexibility of the pupil and autonomic nervous system.

Our results provide novel evidence of the increased prevalence of both parasympathetic and sympathetic autonomic dysfunction in diabetic subjects with and without CAN, as evidenced by a significant reduction in the pupil-iris ratios and longer latency to start of constriction at the 10^th ^flash when compared with healthy volunteers. This shows that dynamic pupillometry may be a valuable tool for the early detection of these abnormalities. Failure to show a statistically significant difference between the diabetes groups in the second experiment may be accounted to the small number of subjects in the CAN group. There is a need for larger as well as prospective studies to better understand this relationship.

Dynamic pupillometry is a simple, inexpensive, and quick technique that requires minimal specialist training. It has the other added advantage over the battery of cardiovascular tests currently in use, in that it is not reliant on active subject participation and compliance. This study also suggests that it is a more sensitive measure of autonomic dysfunction than the conventional autonomic function tests. It also suggests that pupillary autonomic dysfunction occurs early, before a more generalized impairment of the autonomic nervous system, in diabetes.

The pupil-iris ratio in darkness and the pupil reflex amplitude seem to be the dynamic pupillometry parameters most affected by alterations in the autonomic nervous system. Thus, they might be useful in detecting early autonomic dysfunction in high-risk groups.

## Conclusions

In summary, dynamic pupillometry results indicate that this simple and non inexpensive approach can be used as an aiding tool during the clinical examination carried out in the general or specialized clinical settings. This method has the potential to significantly improve the outcome in detecting the early onset of autonomic dysfunction in subjects with diabetes mellitus.

## Competing interests

The authors declare that they have no competing interests.

## Authors' contributions

GLF, JLBM, HRG carried out the study, designed the study concept from a biomedical engineering perspective, designed the instrumentation, carried out the numerical investigations and image processing of pupil images, analyzed the data, and prepared the manuscript. RAG, SRH, ST designed the study concept from a clinical perspective, selected patients to take part in the study, supervised the work, and reviewed the manuscript. FKS designed the instrumentation and developed the algorithms for image processing. All authors have read and approved the final manuscript.
